# Real‐World Comparison of High‐Efficacy Versus Non‐High‐Efficacy Therapies in Multiple Sclerosis

**DOI:** 10.1002/acn3.70130

**Published:** 2025-07-16

**Authors:** Sarmad Al‐Araji, Marcello Moccia, Alessia Bianchi, Charmaine Yam, Weaam Hamed, Suraya Mohamud, Alan J. Thompson, Frederik Barkhof, Ahmed T. Toosy, Olga Ciccarelli

**Affiliations:** ^1^ Queen Square MS Centre, Department of Neuroinflammation UCL Queen Square Institute of Neurology London UK; ^2^ Department of Neurosciences University Hospitals Coventry and Warwickshire Coventry UK; ^3^ Department of Molecular Medicine and Medical Biotechnology Federico II University of Naples Naples Italy; ^4^ Cleveland Clinic London Neurosciences Institute London UK; ^5^ National Hospital for Neurology and Neurosurgery London UK; ^6^ National Institute for Health and Care Research (NIHR) University College London Hospitals (UCLH) Biomedical Research Centre London UK; ^7^ Centre for Medical Image Computing, Department of Medical Physics and Biomedical Engineering University College London London UK; ^8^ Department of Radiology and Nuclear Medicine VU University Medical Centre Amsterdam the Netherlands

**Keywords:** high efficacy therapies, multiple sclerosis, real‐world comparison

## Abstract

**Objective:**

The choice of the first disease modifying treatment (DMT) in multiple sclerosis (MS) is a topic of great interest, and whether high‐efficacy DMTs should be the first choice remains debated. We compared treatment outcomes (no evidence of disease activity [NEDA] and its components) between treatment‐naïve relapsing–remitting MS (RRMS) patients commencing high‐efficacy therapies (HET) and non‐high‐efficacy therapies (non‐HET), using propensity score matching.

**Methods:**

This is an observational prospective study of two real‐world, single‐centre, longitudinal cohorts: (1) Relapsing–remitting MS (RRMS) patients initiated dimethyl fumarate, fingolimod, glatiramer acetate and natalizumab between 2002 and 2020; (2) RRMS patients initiated ocrelizumab between 2019 and 2021. We selected treatment‐naïve patients and had at least 2 years of follow‐up. We compared the two groups at years 1 and 2 using Cox and Logistic regression models as appropriate.

**Results:**

After propensity score matching, we included 448 patients: 110 HET and 338 non‐HET. The probability of losing NEDA was 57% and 39% lower in the HET group at year 1 and 2 (HR = 0.43; 95% CI = 0.35, 0.52; *p* < 0.01 and HR = 0.61; 95% CI = 0.45, 0.84; *p* < 0.01, respectively). The probability of relapse in the HET group was 94% and 71% lower at year 1 and 2 (OR = 0.06; 95% CI = 0.01, 0.28; *p* < 0.01 and OR = 0.29; 95% CI = 0.10, 0.84; *p* < 0.02, respectively). The EDSS in the HET group was 30% and 18% lower at year 1 and 2 (Coeff = −0.30; 95% CI = −0.42, −0.18; *p* < 0.01 and Coeff = −0.16; 95% CI = −0.34, 0.02; *p* < 0.09, respectively). The probability of MRI activity in the HET group was 82% lower at year 1 (OR = 0.18; 95% CI = 0.04, 0.86; *p* < 0.03).

**Interpretation:**

This study demonstrated that treatment‐naïve RRMS patients should be considered for high‐efficacy therapies based on a greater suppression of disease activity at 2 years.

**Summary for social media**

- Choosing the first disease modifying therapy (DMT) in multiple sclerosis (MS) is a topic of great interest for both patients and clinicians, and whether high‐efficacy DMTs should be the first choice remains debated.
- In this real‐world, prospective, single‐centre, cohort of 853 treatment‐naïve relapsing remitting MS patients, those who started high‐efficacy treatment had lower probability of disease activity, clinically and radiologically, at year 1 and 2.
- This study showed that the first choice of treatment in MS patients should include high‐efficacy DMTs based on a greater suppression of disease activity at 2 years, without significant safety concerns.
- This study may encourage clinicians to consider commencing high‐efficay therapies early for patients initiating their first MS treatment.

## 1 Introduction

Multiple sclerosis (MS) is a chronic and progressive neuroinflammatory condition affecting the central nervous system, characterised by heterogeneous clinical manifestations and disease course [1]. MS is the most common cause of serious physical disability in adults of working age and can have a profound impact on quality of life and employment [2, 3].

There have been more than fifteen disease modifying therapies (DMTs) approved for the management of relapsing–remitting MS (RRMS), since the approval of Interferons in 1996 [4]. DMTs can be divided into non‐high‐efficacy therapies (non‐HET), with a much better‐defined safety profile, and high‐efficacy therapies (HET), with potentially higher risk of serious side effects [5].

The choice of the first DMT in treatment‐naïve MS patients is a topic of great interest for both patients and clinicians, and whether HET should be preferred to a non‐HET remains a topic for debate in the MS community. The recent ABN guidelines have recommended that HET should be considered as the first option in eligible patients [6]. Previous international guidelines recommended HET for highly active MS (ECTRIMS [European Committee for Treatment and Research in Multiple Sclerosis]/EAN [European Academy of Neurology] and American Academy of Neurology [AAN] guidelines) [7, 8].

Real‐world cohorts have shown that DMTs significantly reduced the risk of reaching disability milestones in the long‐term [9, 10]. When real‐world cohorts have been used to compare the efficacy of DMTs, they have rarely included statistical matching techniques, which are necessary to account for systematic differences in baseline characteristics between patients initiating different DMTs when estimating the effectiveness of DMTs [11, 12]. However, when they did use them, there were other shortcomings: (1) They studied only clinical relapses and disability, and not MRI activity, (2) They combined treatment‐naïve patients together with patients previously treated with other DMTs and (3) They performed single comparisons of two DMTs [13, 14, 15, 16].

Head‐to‐head randomised clinical trials (RCTs) offer the gold standard design for estimating the effects of treatments. However, they have made only one‐to‐one comparisons, and included patients switching therapies together with treatment naïve patients; additionally, the trial conditions are not often generalisable and, for example, they do not include patients with multiple co‐morbidities. There is evidence from single‐arm, open‐label studies that treatment‐naïve patients maintain low disease activity when they initiate HET, but they have been not compared to other therapies, and are affected by ‘selection bias’ [17].

No evidence of disease activity (NEDA) has been used as a tool for measuring the efficacy of DMTs. NEDA (also known as NEDA3) is achieved if there are no new clinical relapses, new activity on magnetic resonance imaging (MRI) and signs of clinical disease progression, measured by expanded disability status scale (EDSS) over a defined period of time [18]. Although NEDA has its own limitations as a tool to measure the heterogeneity of MS worsening, poor treatment response in the early stages of MS has significant prognostic value [19, 20].

We aimed to compare treatment outcomes (numbers of relapses, disability worsening, MRI activity and NEDA status at 1 and 2 years) between treatment‐naïve patients commencing HET versus non‐HET, whereas accounting for systematic differences in baseline characteristics of the two groups with propensity score matching.

## 2 Methods

### 2.1 Study Design and Participants

This is an analysis of two real‐world, single‐centre (Queen Square MS Centre, University College London, UK) longitudinal cohorts, which were combined:

1. A cohort of all consecutive RRMS patients who commenced dimethyl fumarate, fingolimod, glatiramer acetate, and natalizumab between 2002 and 2021. The clinical and radiological data for this cohort were recorded prospectively at patients' visits by consultant MS specialists and collected by the first author through the hospital electronic health records.
2. A prospective cohort of consecutive RRMS patients who initiated ocrelizumab between 2019 and 2021. Patients in this cohort were assessed at the initiation of ocrelizumab and then at least annually, as part of the POINT‐MS study.

The inclusion criteria for the present study were

1. Diagnosis of RRMS,
2. Age ≥ 18 years,
3. First DMT prescription,
4. Availability of at least 2 years of follow‐up (or reaching study outcomes before 2 years of follow‐up) with clinical and radiological variables.

We excluded MS patients with a previous history of DMT use and progressive (primary and secondary) MS patients.

Clinical variables were collected at study entry (within a maximum of 3 months from the first dose or infusion of DMT), and at year 1 and year 2. Patients who discontinued DMT prior to years 1 and 2 because of disease activity contributed to the pool of patients available for NEDA assessment. On the contrary, patients who stopped treatment due to other reasons, such as side effects, family planning, or those who moved to a different location (and therefore to a different MS service) were excluded from analyses at year 1, if they stopped treatment within the first 6 months, and at year 2, if they stopped treatment within 18 months.

The following variables were collected at baseline (i.e., first treatment initiation): age, sex (as recorded on the health records), ethnicity (self‐identified), age at MS onset (which was used to calculate disease duration), disease duration (time from MS clinical onset to treatment initiation), number of relapses in the previous 12 months, Expanded‐Disability Status Scale (EDSS), and brain MRI activity (including number of new and/or enlarging T2 and gadolinium‐enhancing [Gd+] T1 lesions (also referred to as ‘active lesions‘) compared with the previous scan within the previous 24 months, as described in the neuro‐radiology reports).

We acquired the MRIs according to local, standard‐of‐care protocols in the national health service over the last 20 years. As expected, there was a variety of sequences and parameters that had changed over time, and many different protocols were used. This may have introduced bias in terms of the ability to identify lesions, but we used clinical neuro‐radiologist reports for the number of lesions identified and compared them to previous scans with a similar protocol.

The outcomes collected at year 1 and 2 were: (i) Number of patients who relapsed from baseline to year 1, and from year 1 to year 2; (ii) Number of patients who had EDSS progression, defined as an increase of either ≥ 0.5 points for patients with a baseline EDSS score ≥ 5.5, ≥ 1.0 point for those with a baseline EDSS score of 1.0 and 5.0, and ≥ 1.5 points for those with a baseline EDSS score of 0; (iii) Number of patients who experienced MRI activity on brain MRI from baseline to year 1, and from year 1 to year 2; (iv) NEDA, which implies NEDA3 throughout this study, is defined as the absence of relapses, EDSS progression and/or active MRI lesions; this was calculated at year 1, at year 2, and between year 1 and 2.

The study of these two cohorts was approved by the research ethics committee (19/WA/0157 and 23/WS/0008).

### 2.2 Statistical Analysis

Mean (and standard deviation [SD]), median (and range) and number (and percent) were calculated for different study variables, as appropriate. Differences in demographic, clinical and radiological characteristics at treatment initiation between non‐high‐efficacy (dimethyl fumarate, glatiramer acetate, fingolimod) and high‐efficacy DMTs (natalizumab, ocrelizumab) were evaluated using *t*‐test, chi‐square test and Fisher's exact test, as appropriate.

We used inverse‐probability weighting regression analysis to estimate the probability of treatment allocation (high‐efficacy therapy (HET) vs. non‐high‐efficacy therapy (non‐HET)), based on age, sex, ethnicity, disease duration, relapses in the year before treatment initiation, EDSS at treatment initiation and active MRI lesions at treatment initiation. Based on propensity scores calculated above, patients were matched by nearest neighbour, within 0.1 SD of the propensity score. Then, we compared HET and non‐HET using logistic regression (probability of relapse at year 1 and 2, probability of new MRI lesions at year 1 and 2), linear regression (EDSS changes at year 1 and 2), and Cox regression models (probability of NEDA at year 1 and 2, and between year 1 and 2).

Results were reported as coefficients (Coeff), hazard ratio (HR), odds ratio (OR), 95% confidence interval (95% CI), and *p* values, as appropriate. Distribution of variables and residuals was checked using both graphical and statistical methods. Statistical analyses were performed using Stata 15.0 (StataCorp, College Station, TX, USA). Results were considered statistically significant if *p* < 0.05.

### 2.3 Power Calculation

We performed the power calculation based on our matched sample of 689 patients on non‐HET and 164 on HET to understand what statistical power we would have had with our sample size. Findings showed that the sample of 689 versus 164 patients would have been sufficient to detect a 1.3% difference in any of the treatment outcomes between groups, using regression models, with a 5% alpha and 90% power.

## 3 Results

**FIGURE 1:**
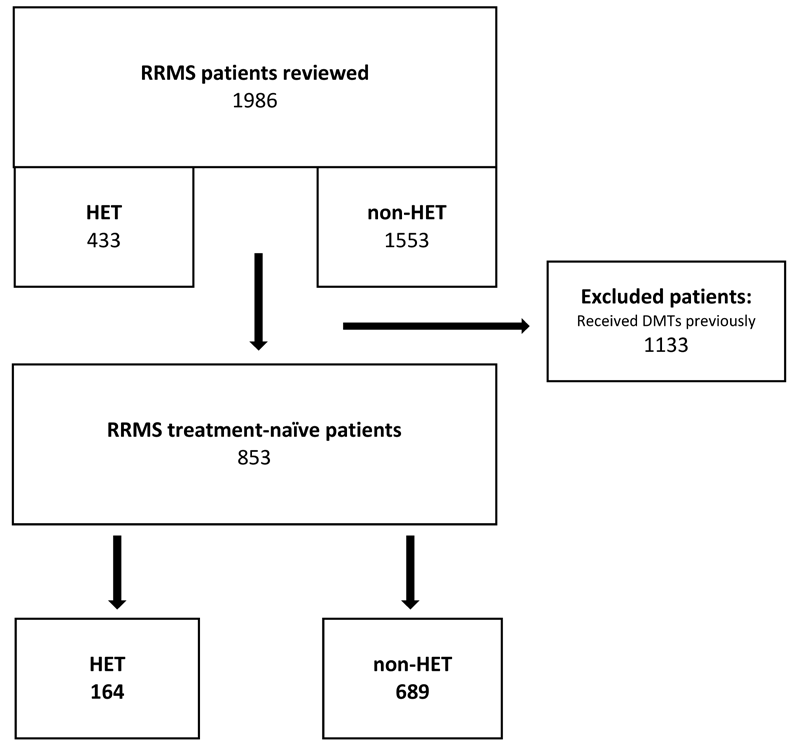
Study population flow chart. A flow chart of the patients with relapsing remitting multiple sclerosis (RRMS) who initiated high‐efficacy therapy (HET) or non‐high‐efficacy therapy (non‐HET) at our centre.

A flow chart of patients is shown in Figure 1. We identified 1986 patients in our whole cohort: 433 on HET and 1553 on non‐HET. Table S1 shows the demographics, clinical and radiological characteristics of the whole cohort. We found 853 treatment‐naïve RRMS patients who commenced their first DMT: 164 started HET and 689 non‐HET. Table S2 shows the demographics, clinical and radiological characteristics of the treatment‐naïve cohort. Out of 853 patients, 735 (164 HET and 649 non‐HET) were eligible for NEDA assessment at year 1 and 2. Table S3 shows the clinical outcomes of the treatment‐naïve cohort at year 1 and 2. This table demonstrates that the HET group has better outcomes than the non‐HET group at year 1 and 2. Although ocrelizumab has the larger number of patients, both natalizumab and ocrelizumab have shown similarly better outcomes compared to the non‐HET group. The number of patients losing NEDA in the natalizumab and ocrelizumab group was 8.0% and 5.1%, respectively, whereas the number of patients losing NEDA in the glatiramer acetate, dimethyl fumarate and fingolimod group was 24.4%, 15.0% and 41.7%, respectively.

After propensity score matching, we included 448 treatment‐naïve patients (110 on HET and 338 on non‐HET). Demographics, clinical and radiological characteristics at treatment initiation for this cohort are shown in Table 1. Clinical and radiological outcomes at year 1 and 2 are reported in Table 2.

**TABLE 1:** Demographic, clinical and radiological characteristics at treatment initiation after propensity score matching.

	Non‐high‐efficacy DMTs	High‐efficacy DMTs	*p*
Number of patients included after propensity score matching	338	110	
Sex: Female (%)	247 (73)	79 (72)	0.44
Ethnicity (self‐identified)			
White	282 (83%)	97 (88%)	0.65
Non‐White	56 (17%)	13 (12%)	
Mean age at study entry (years)	38.2 ± 9.7	36.9 ± 10.1	0.20
Mean disease durations (years)	6.5 ± 4.8	5.9 ± 6.0	0.24
No. of relapses in previous 12 months (mean ± SD)	1.1 ± 0.8	1.2 ± 0.8	0.37
Mean EDSS at baseline	2.7 ± 1.7	2.6 ± 1.7	0.76
No. of new T2 and enlarging lesions (compared with the previous scan within the previous 24 months) and Gd + lesions on brain MRI at baseline	0.5 ± 1.3	0.6 ± 1.4	0.51

*Note: p* values from *t*‐test, chi‐square test and Fisher's exact test, as appropriate, comparing demographic, clinical and radiological characteristics between patients commencing non‐high‐efficacy therapy (non‐HET) versus high‐efficacy therapy (HET) as their first disease modifying treatment.

Abbreviations: DMT, disease modifying therapy; EDSS, expanded disability status scale; Gd+, gadolinium enhancing.

**TABLE 2:** Relapses, MRI activity, EDSS progression and NEDA at 1 and 2 years after propensity score matching.

DMT	Non‐HET	HET		95% CI		*p*
	338	110		Lower	Upper	
Year 1						
Number (%) of patients with NEDA	243 (71.9%)	101 (91.8%)	HR = 0.43	0.35	0.52	< 0.01
Number (%) of patients with relapses	68 (21.0%)	2 (1.8%)	OR = 0.06	0.01	0.28	< 0.01
Number (%) of patients with MRI activity	13 (11.9%)	2 (2.5%)	OR = 0.18	0.04	0.86	0.03
EDSS (mean ± SD)	2.8 ± 1.8	2.4 ± 1.8	Coeff = −0.30	−0.42	−0.18	< 0.01
Year 2						
Number (%) of patients with NEDA	181 (53.7%)	81 (73.6%)	HR = 0.61	0.45	0.84	< 0.01
Number (%) of patients with relapses	56 (19.5%)	4 (6.6%)	OR = 0.29	0.10	0.84	0.02
Number (%) of patients with MRI activity	23 (21.7%)	0 (0%)	—	—	—	—
EDSS (mean ± SD)	2.8 ± 1.9	2.9 ± 2.1	Coeff = −0.16	−0.34	0.02	0.09
Number (%) of patients with NEDA between year 1 and 2	129 (38.2%)	58 (52.8%)	HR = 0.72	0.53	0.97	0.03

*Note:* Coefficients (Coeff), hazard ratio (HR), odds ratio (OR), 95% confidence interval (95% CI), and *p* values from logistic regression, linear regression and Cox regression models, as appropriate, comparing patients commencing non‐high‐efficacy therapy (non‐HET) versus high‐efficacy therapy (HET) as their first disease modifying treatment.

Abbreviations: EDSS, expanded disability status scale; NEDA, no evidence of disease activity.

Table S4 shows the reasons patients stopped treatment early and therefore were not eligible for NEDA assessment. Only patients who initiated non‐HET stopped early, with the majority of patients stopping dimethyl fumarate and glatiramer acetate due to side effects.

### 3.1 Differences in Outcomes Between HET and Non‐HET at 1 and 2 Years

**FIGURE 2:**
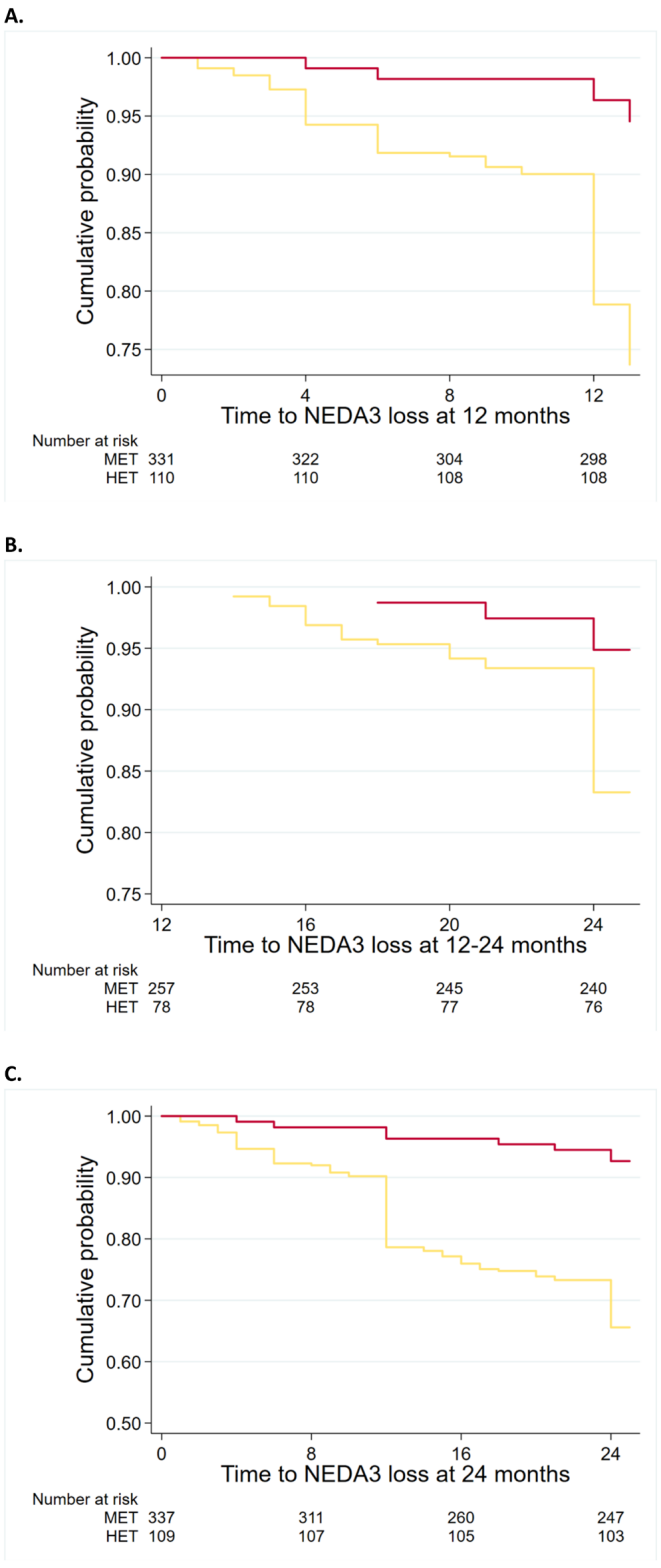
NEDA for patients commencing on HET or non‐HET. Kaplan–Meier Curves estimate the rates of no evidence of disease activity (NEDA, also known as NEDA3) loss in high‐efficacy therapy (HET) [red] or non‐high‐efficacy therapy (non‐HET) [yellow] at 1 year (A), between 1 and 2 years (B), and at 2 years (C). Note that the values of the measurements shown here are the absolute values and are unadjusted.

When compared with non‐HET, the probability of losing NEDA in the HET group was 57% lower at year 1 (HR = 0.43; 95% CI = 0.35, 0.52; *p* < 0.01), 39% lower at year 2 versus baseline (HR = 0.61; 95% CI = 0.45, 0.84; *p* < 0.01) and 28% lower between year 1 and year 2 (HR = 0.72; 95% CI = 0.53, 0.97; *p* = 0.03) (Figure 2).

**FIGURE 3:**
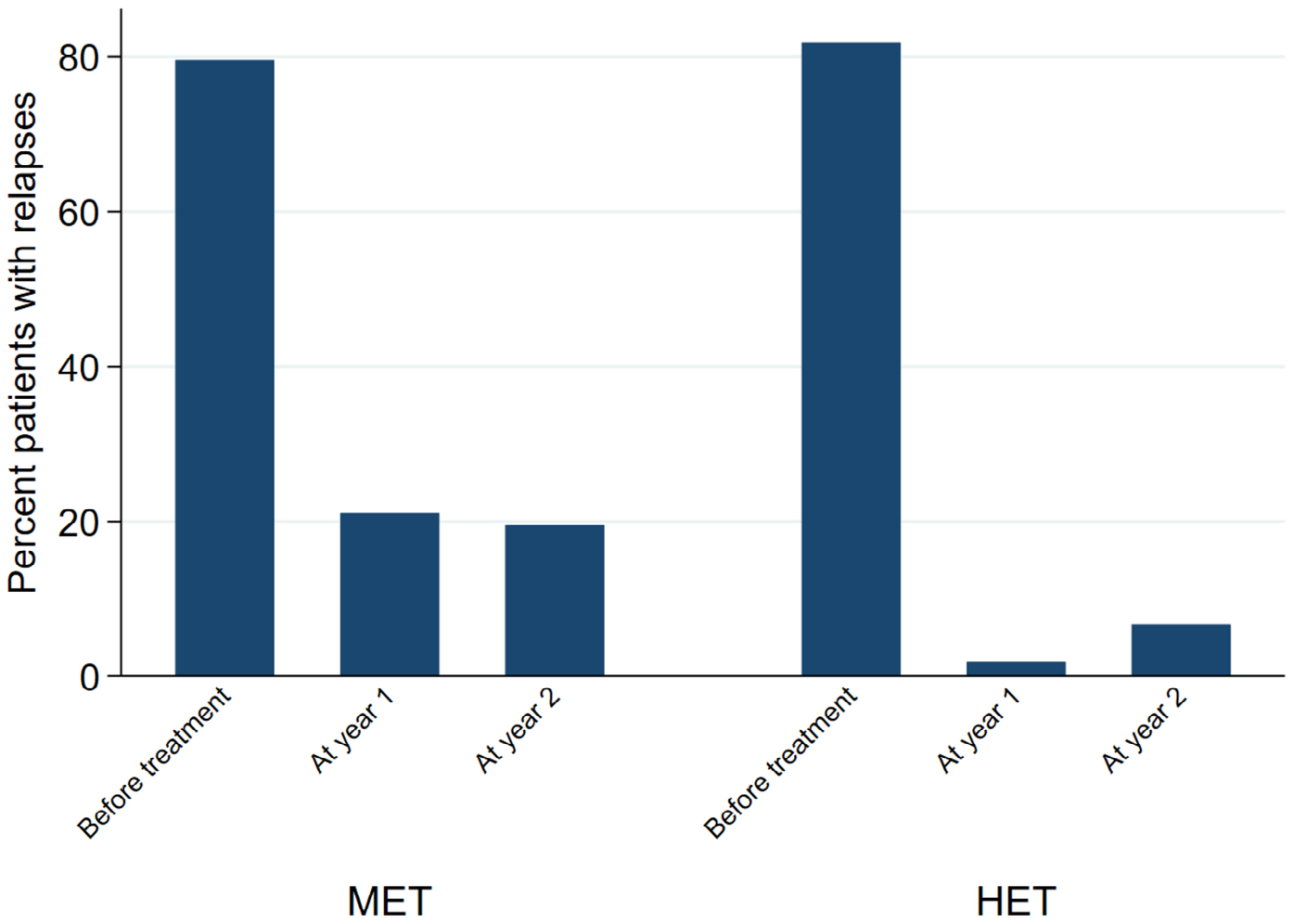
Relapses for patients commencing on HET or non‐HET. Bar graph shows the percent number of patients with relapses in the 12 months before starting treatment (baseline), and at year 1 and 2 after starting high‐efficacy therapy (HET) or non‐high‐efficacy therapy (non‐HET). Note that the values of the measurements shown here are the absolute values and are unadjusted.

**FIGURE 4:**
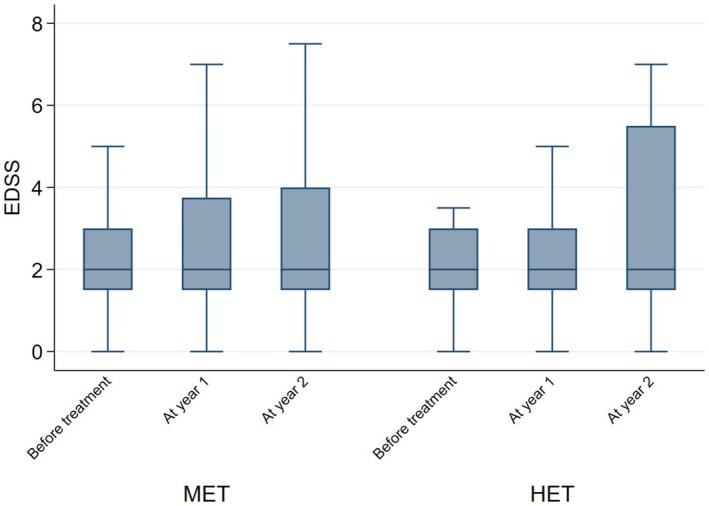
EDSS for patients commencing on HET or non‐HET. Box‐and‐whisker plot shows expanded disability status scale (EDSS) before starting treatment (baseline), and at year 1 and 2 after starting high‐efficacy therapy (HET) or non‐high‐efficacy therapy (non‐HET). Note that the values of the measurements shown here are the absolute values and are unadjusted.

**FIGURE 5:**
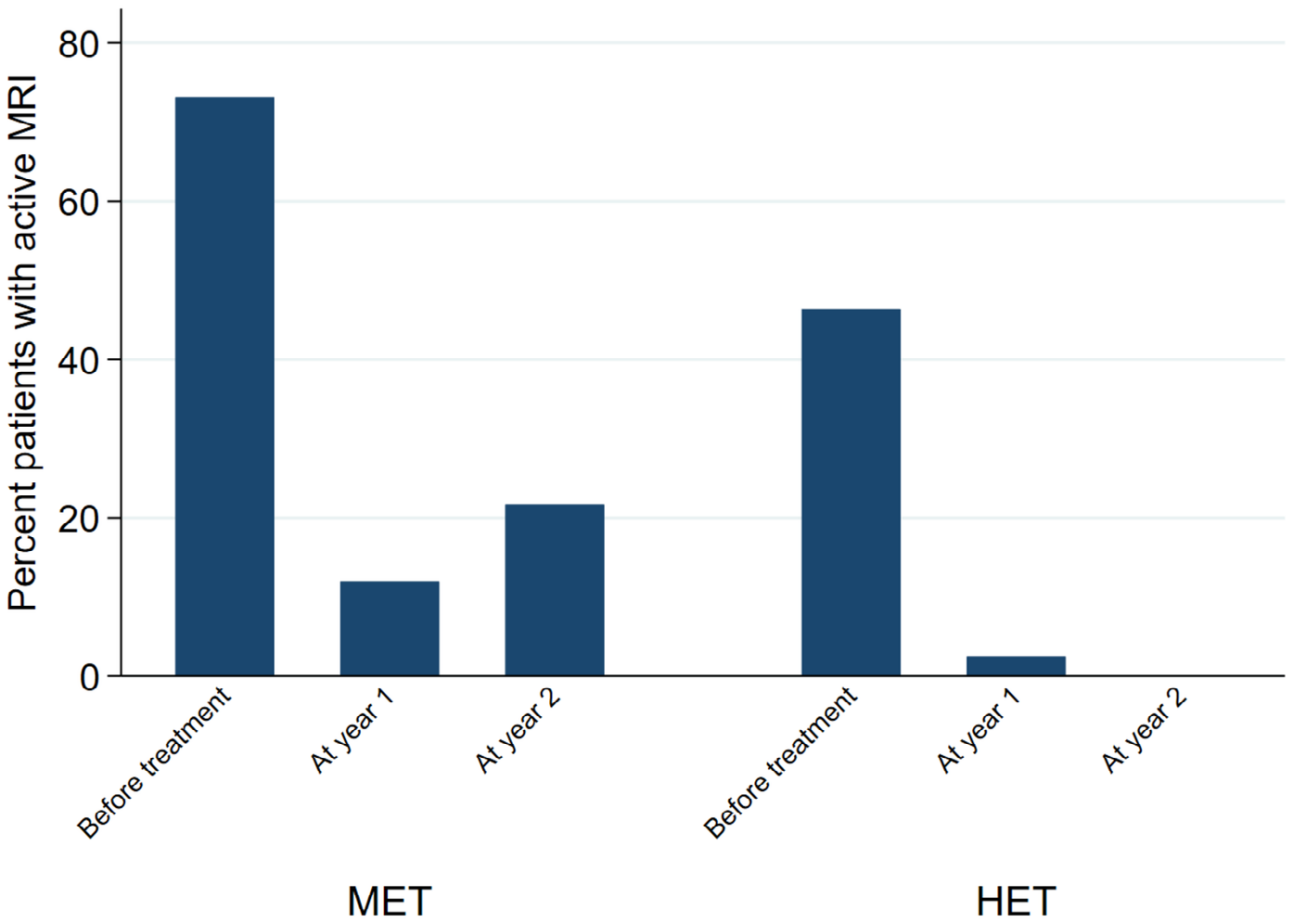
MRI activity for patients commencing on HET or non‐HET. Bar graph shows the percent of patients with new/Gadolinium enhancing lesions before starting treatment (baseline), and after 1 and 2 years after commencing high‐efficacy therapy (HET) or non‐high‐efficacy therapy (non‐HET). Note that the values of the measurements shown here are the absolute values and are unadjusted.

When looking at the individual NEDA components, the findings were similar. When compared with non‐HET, the probability of relapse in the HET group was 94% lower at year 1 (OR = 0.06; 95% CI = 0.01, 0.28; *p* < 0.01), and 71% lower at year 2 (OR = 0.29; 95% CI = 0.10, 0.84; *p* = 0.02) (Figure 3). EDSS at year 1 was 30% lower in the HET group (2.43 ± 1.88) (Coeff = −0.30; 95% CI = ‐0.42, −0.18; *p* < 0.01) compared with non‐HET (2.80 ± 1.87), whereas no difference was detected at year 2 (Coeff = −0.16; 95% CI = ‐0.34, 0.02; *p* = 0.09) (Figure 4). The probability of active MRI was 82% lower at year 1 in the HET group (OR = 0.18; 95% CI = 0.04, 0.86; *p* = 0.03) compared with non‐HET. A statistical analysis was not possible at year 2 versus year 1 due to the lack of active MRI in the HET group; in the non‐HET group the percentage of patients with MRI activity was 21.7% (Figure 5).

## 4 Discussion

In this single centre, real‐world prospective observational study, we evaluated the short and medium‐term treatment response in treatment‐naïve RRMS patients who commenced their first therapy with HET or non‐HET. Using a propensity score‐based matching, we compared RRMS patients with similar demographics and baseline disease characteristics. We found that the probability of relapse, EDSS progression and MRI activity was lower in the HET group compared with the non‐HET group. Collectively, the probability of losing NEDA was lower in the HET group compared with the non‐HET group. This is due to a more potent effect on disease activity of the high‐efficacy DMTs [20].

The main novelties of our study are that we adjusted for possible differences in treatment allocation driven by different clinical characteristics, included several medications used in the real world, and analyzed MRI treatment outcomes. The strength of our study is the well defined study population that reflects routine clinical practice in a Universal Health Care system: MS patients are followed by MS neurologists at a public neuroscience center and have access to all licenced DMTs. We aimed to minimise selection bias by including all consecutive patients and describing reasons for exclusion.

Subgroup analyses and open‐label extensions of RCTs on ocrelizumab, cladribine and natalizumab have confirmed their high efficacy in controlling disease activity when initiated early [17, 21, 22, 23]. However, single‐arm, open‐label studies are affected by selection bias, as they recruit participants who have responded (and continue to respond) to the medication. Five real‐world retrospective studies, which have investigated non‐high‐efficacy therapy versus high‐efficacy therapy strategies, suggested an association of high‐efficacy therapy with a lower risk of clinical relapses, a lower mean change in EDSS score at 5–10 years, a higher median time to sustained accumulation of disability and a lower risk of conversion to secondary progressive MS, compared with starting non‐high‐efficacy agents [24, 25, 26, 27, 28, 29]. However, these studies have lacked MRI data, and considering the baseline MRI features are a crucial prognostic factor to guide the first treatment choice, we included MRI data at baseline as a covariate for the patient propensity score‐matching procedure and as a measure of outcome that could trigger treatment escalation.

Some clinicians are advocating the initiation of a non‐HET in treatment‐naïve patients, with a timely switch to a more potent agent which can control breakthrough disease in the case of an inadequate response to the first‐line DMTs, on the basis of the available evidence and international consensus guidelines: (1) The European (ECTRIMS/EAN) guidelines of 2018 suggest deciding MS therapy depends on patient characteristics, disease severity, safety profile and drug accessibility and advise escalating treatment in the case of disease activity while on non‐high‐efficacy DMT [7]. (2) The AAN guidelines recommend that patients with a highly active disease should be treated with high‐efficacy DMT [8]. Neither the AAN nor the European guidelines recommend a specific treatment strategy. (3) The most recent guidelines from the ABN recommend that high‐efficacy therapy should be considered as the first option in eligible patients [6].

There are some limitations in our study. No patients on interferons were included in the non‐HET group and no patients on cladribine or alemtuzumab were included in the HET group. We aim to include all therapies as we collect data in due course, but therapies such as interferons and alemtuzumab are currently rarely offered. Also, until recently, cladribine could only be prescribed to patients with highly active and rapidly evolving severe MS, and would require a longer follow‐up than 2 years. Since fingolimod is a second‐line DMT in England, only a small number of patients were included in this study who started fingolimod as a first‐line therapy for a variety of reasons including needle phobia and gastrointestinal conditions preventing them from commencing other first‐line therapies. Considering studies have shown that fingolimod has a similar efficacy profile to dimethyl fumarate, we have categorised patients starting fingolimod in the non‐HET group in our cohort [30, 31]. We have included several therapies in the non‐HET group which may have varying degrees of efficacy; however, real‐life observational studies have not shown significant differences between dimethyl fumarate and fingolimod and between interferon and glatiramer acetate [16, 32, 33]. The number of patients on each disease modifying drug was too small to allow a more granular comparison between different therapies.

The single‐centre design of this study may limit generalisability. However, our cohort is derived from a tertiary referral hospital, with a large catchment area, including a large multi‐national and multi‐ethnical population. Another limitation is that we evaluated patients over a period of 2 years, which is the common duration of clinical trials, but the long‐term effects of treatments will require longer follow‐ups; future studies will assess the outcomes of these cohorts in the long‐term. This is especially the case for EDSS; we showed EDSS reduction at year 1 in HET (possibly reflecting rapid anti‐inflammatory activity) but failed to find any further changes at year 2, with the activity on neurodegenerative aspects of progression requiring longer observation time. EDSS is the most used parameter for the assessment of neurological impairment and disease progression, as it is easily performed and intuitively interpreted by any clinician familiar with the standard neurological examination. However, it is important to note that one of the main limitations of EDSS is that it is regarded as heavily weighted on mobility and not very sensitive to cognitive impairment or upper limb dysfunction [34].

Different DMTs have been offered to patients at different time periods over the last two decades, depending on treatment availability and, therefore, a possible confounder leading to patients' inhomogeneity is the different treatment allocation over time (i.e., ocrelizumab became available in the UK in 2019), which, however, may have affected both groups of patients. Finally, we only included MRI brain analysis and future work will aim to include spinal cord MRI scans, as well as data of other therapies with longer‐term outcomes.

The propensity score matching cannot control for unknown confounders (and for any covariate that was not measured and/or not included in propensity score calculation). Therefore, results of two ongoing prospective, multicentre, randomised, pragmatic trials comparing early high‐efficacy DMT with escalation approach may provide useful information (the TRaditional versus Early Aggressive Therapy for MS (TREAT‐MS, ClinicalTrials.gov no. NCT03500328) and the Determining the Effectiveness of Early Intensive versus Escalation Approaches for the Treatment of RRMS (DELIVER‐MS, ClinicalTrials.gov no. NCT03535298)). However, the advantage of real‐world cohorts is that they may include patients older than 55 years and with multiple co‐morbidities, who are often excluded from trials.

In summary, the analysis of a real‐world cohort using propensity score matching showed that the use of highly effective DMTs increases the probability of achieving a status of no disease activity at 1 and 2 years, with no concerns related to safety, suggesting that high‐efficacy DMTs should be considered as the first option for treatment‐naïve patients.

## Author Contributions

Al‐Araji had full access to all of the data in the study and takes responsibility for the integrity of the data and the accuracy of the data analysis. Concept and design: Al‐Araji, Ciccarelli. Acquisition, analysis, or interpretation of data: All authors. Drafting of the manuscript: Al‐Araji, Moccia, Ciccarelli. Critical revision of the manuscript for important intellectual content: Al‐Araji, Moccia, Thompson, Barkhof, Ciccarelli. Statistical analysis: Al‐Araji, Moccia. Obtained funding: Ciccarelli. Administrative, technical, or material support: Al‐Araji, Moccia, Ciccarelli. Supervision: Ciccarelli.

## Conflicts of Interest

Sarmad Al‐Araji: received sponsorship from Sanofi to attend ECTRIMS and speaker honoraria from Roche. Marcello Moccia: funded by the MUR PNRR Extended Partnership (MNESYS no. PE00000006). In the last 3 years, he has received honoraria from Biogen, BMS Celgene, Ipsen, Merck, Novartis, Roche and Sanofi‐Genzyme. Alessia Bianchi has received a research grant from the Italian Society of Neurology and is an ECTRIMS‐MAGNIMS Fellow. Charmaine Yam is a Cleveland Clinic London PhD Neuroscience Fellow. Weaam Hamed: Nothing to report. Suraya Mohamud: Nothing to report. Alan J. Thompson: receives an honorarium from SAGE Publishers as Editor‐in‐Chief of Multiple Sclerosis Journal; has received support from UCL/UCLH NIHR Biomedical Research Centre; acted as co‐chair at UCL‐Eisai Steering Committee drug discovery collaboration (fees paid to the institution). Ahmed T. Toosy: speaker honoraria from Merck, Biomedia, Sereno Symposia International Foundation, Bayer and At the Limits and meeting expenses from Merck, Biogen Idec and Novartis. He was the UK PI for two clinical trials sponsored by MEDDAY pharmaceutical company (MD1003 in optic neuropathy [MS‐ON‐NCT02220244] and progressive MS [MS‐SPI2—NCT02936037]). He has been supported by recent grants from the MRC (MR/S026088/1), NIHR BRC (541/CAP/OC/818837) and RoseTrees Trust (A1332 and PGL21/10079). He is an associate editor for Frontiers in Neurology—Neuro‐ophthalmology section and on the editorial board for Neurology and Multiple Sclerosis Journal. Frederik Barkhof: acts as a member of the steering committee or Data Safety Monitoring Board for Biogen, Merck, ATRI/ACTC and Prothena. Consultant for Roche, Celltrion, Rewind Therapeutics, Merck, IXICO, Jansen and Combinostics. Research agreements with Merck, Biogen, GE Healthcare and Roche. Co‐founder and shareholder of Queen Square Analytics LTD. Olga Ciccarelli: NIHR Research Professor (RP‐2017‐08‐ST2‐004); over the last 2 years, member of independent DSMB for Novartis; she gave a teaching talk in a Merck local symposium, and contributed to an Advisory Board for Biogen; she is Deputy Editor of Neurology, for which she receives an honorarium; she has received research grant support from the MS Society of Great Britain and Northern Ireland, the NIHR UCLH Biomedical Research Centre, the Rosetree Trust, the National MS Society and the NIHR‐HTA.

## Supporting information


**Table S1.** Demographic, clinical and radiological characteristics of the whole cohort at treatment initiation.
**Table S2.** Demographic, clinical and radiological characteristics of the treatment‐naïve patients at treatment initiation before propensity score matching.
**Table S3.** Clinical outcomes of the treatment‐naïve patients at year 1 and 2 before propensity score matching.
**Table S4.** Reasons for patients not assessed for NEDA according to type of therapy.

## Data Availability

Tabulated, anonymised data may be shared after appropriate approval is sought.
