# Supplementary material for: Real‐World Comparison of High‐Efficacy Versus Non‐High‐Efficacy Therapies in Multiple Sclerosis
Source: Ann Clin Transl Neurol. 2025 Jul 16;12(10):2077–85. doi: 10.1002/acn3.70130 (PMC12516238; doi:10.1002/acn3.70130)
Supplement: Supplementary file 1 — Table S1. Demographic, clinical and radiological characteristics of the whole cohort at treatment initiation. Table S2. Demographic, clinical and radiological characteristics of the treatment‐naïve patients at treatment initiation before propensity score matching. Table S3. Clinical outcomes of the treatment‐naïve patients at year 1 and 2 before propensity score matching. Table S4. Reasons for patients not assessed for NEDA according to type of therapy. [file ACN3-12-2077-s001.docx]

**Supplementary material:**

**Supplementary Table 1. Demographic, clinical and radiological characteristics of the whole cohort at treatment initiation.**

|  | **Non-high-efficacy DMTs** | | | | **High-efficacy DMTs** | | |
| --- | --- | --- | --- | --- | --- | --- | --- |
| **DMT** | **Glatiramer acetate** | **Dimethyl fumarate** | **Fingolimod** | **Total** | **Natalizumab** | **Ocrelizumab** | **Total** |
| **Number of patients** | 547 | 670 | 336 | 1553 | 177 | 256 | 433 |
| **Sex: Female (%)** | 407  (74) | 483  (72) | 252  (75) | 1142  (74) | 113  (64) | 160  (63) | 273  (63) |
| **Ethnicity – White (%)** | 425  (78) | 478  (71) | 253  (75) | 1156  (79) | 119  (67) | 193  (75) | 312  (72) |
| **Mean age at study entry (years)** | 38.6  ±9.5 | 40.5  ±10.2 | 39.2  ±10.4 | 39.6  ±10.0 | 36.1  ±10.2 | 38.7  ±10.6 | 37.7  ±10.5 |
| **Mean disease durations (years)** | 7.2  ±6.6 | 8.6  ±7.1 | 10.6  ±7.1 | 8.6  ±7.0 | 8.4  ±6.6 | 7.0  ±6.8 | 7.6  ±6.7 |
| **No. of relapses in previous 12 months** | 1.0  ±0.8 | 0.9  ±0.8 | 1.0  ±0.8 | 1.0  ±0.8 | 1.8  ±1.0 | 0.9  ±0.8 | 1.3  ±1.0 |
| **Mean EDSS at baseline** | 2.5  ±1.7 | 2.7  ±1.8 | 3.4  ±1.9 | 2.7  ±1.8 | 4.7  ±2.0 | 2.7  ±1.8 | 3.5  ±2.2 |
| **No. of new MRI lesions at baseline** | 1.5  ±1.9 | 0.8  ±1.4 | 0.7  ±1.7 | 1.0  ±1.7 | 1.9  ±2.1 | 1.4  ±2.1 | 1.3  ±1.0 |

P-values from t-test, chi-square test and Fisher's exact test, as appropriate, comparing demographic, clinical and radiological characteristics between patients commencing non-high-efficacy therapy (non-HET) versus high-efficacy therapy (HET) as their first disease modifying treatment. DMT = disease modifying therapy. EDSS = expanded disability status scale.

**Supplementary Table 2. Demographic, clinical and radiological characteristics of the treatment-naïve patients at treatment initiation before propensity score matching.**

|  | **Non-high-efficacy DMTs** | | | | **High-efficacy DMTs** | | |  |
| --- | --- | --- | --- | --- | --- | --- | --- | --- |
| **DMT** | **Glatiramer acetate** | **Dimethyl fumarate** | **Fingolimod** | **Total** | **Natalizumab** | **Ocrelizumab** | **Total** | **p-values** |
| **Number of patients** | 342 | 335 | 12 | **689** | 26 | 138 | **164** |  |
| **Sex: Female (%)** | 246  (72) | 235 (70%) | 9  (75) | 490  (71) | 18  (69) | 89  (62) | 107  (65) | <0.01 |
| **Ethnicity (%)**  White  Non-White | 272 (80%)  70 (20%) | 220 (65)  115 (34) | 7 (58)  5 (42) | 499 (72)  190 (28) | 17 (65)  9 (35) | 102 (74)  36 (26) | 119 (73)  45 (27) | 0.16 |
| **Mean age at study entry (years)** | 37.9±9.4 | 40.0±10.2 | 29.2±12.9 | 38.8±10.0 | 31.3±7.8 | 38.6±11.2 | 37.4±11.0 | 0.21 |
| **Mean disease durations (years)** | 5.5±5.3 | 6.3±6.4 | 5.3±6.2 | 5.9±5.9 | 4.2±3.3 | 4.8±6.5 | 4.7±6.1 | 0.01 |
| **Mean No. of relapses in previous 12 months** | 1.2±0.8 | 1.1±0.8 | 1.8±0.8 | 1.2±0.8 | 2.3±1.1 | 1.2±0.8 | 1.6±0.9 | 0.02 |
| **Mean EDSS at baseline** | 2.4±1.6 | 2.2±1.5 | 2.1±1.6 | 2.3±1.6 | 3.8±2.5 | 2.3±1.5 | 2.5±1.8 | 0.10 |
| **No. of new MRI lesions at baseline** | 1.7±2.0 | 1.0±1.5 | 1.4±1.8 | 1.3±1.8 | 2.9±3.0 | 1.4±2.4 | 1.6±2.5 | <0.01 |

P-values from t-test, chi-square test and Fisher’s exact test, as appropriate, comparing demographic, clinical and radiological characteristics between patients commencing non-high-efficacy therapy (non-HET) versus high-efficacy therapy (HET) as their first disease modifying treatment. DMT = disease modifying therapy. EDSS = expanded disability status scale.

**Supplementary Table 3. Clinical outcomes of the treatment-naïve patients at year 1 and 2 before propensity score matching.**

|  | **Non-high-efficacy DMTs** | | | | **High-efficacy DMTs** | | |
| --- | --- | --- | --- | --- | --- | --- | --- |
| **DMT** | **Glatiramer acetate** | **Dimethyl fumarate** | **Fingolimod** | **Total** | **Natalizumab** | **Ocrelizumab** | **Total** |
| **Number of patients** | 324 | 313 | 12 | 649 | 26 | 138 | 164 |
| **Year 1** | | | | | | | |
| **Number (%) of patients with relapses** | 76  (23.5) | 37  (11.8) | 1  (8.3) | 114 (17.6) | 1  (3.8) | 3  (2.2) | 4 (2.4) |
| **Mean EDSS** | 2.4  ±1.7 | 2.2  ±1.6 | 2.1  ±1.6 | 2.3  ±1.6 | 3.7  ±2.5 | 2.0  ±1.5 | 2.3  ±1.8 |
| **Number (%) of patients with EDSS progression** | 17  (5.3) | 12  (3.8) | 0 | 29 (4.5) | 1  (3.8) | 8  (6.0) | 9 (5.5) |
| **Number (%) of patients with active MRI brain compared to baseline*** | 26  (8.0) | 31  (9.9) | 0 | 57 (8.8) | 0 | 3  (2.2) | 3 (1.8) |
| **Number (%) of patients who lost NEDA** | 92  (28.4) | 58  (18.5) | 1  (8.3) | 151 (23.3) | 2  (8.0) | 9  (6.5) | 11 (6.7) |
| **Year 2** | | | | | | | |
| **Number (%) of patients with relapses** | 52  (16.0) | 22  (7.0) | 4  (33.3) | 78 (12.0) | 2  (8.0) | 4  (2.9) | 6 (3.7) |
| **Mean EDSS** | 2.6  ±1.8 | 2.2  ±1.5 | 2.1  ±1.6 | 2.4  ±1.7 | 3.6  ±2.4 | 2.3  ±1.6 | 2.7  ±2.0 |
| **Number (%) of patients with EDSS progression** | 18  (5.6) | 3  (1.0) | 0 | 21 (3.2) | 0 | 4  (2.9) | 4 (2.4) |
| **Number (%) of patients with active MRI brain*** | 27  (8.3) | 28  (9.0) | 2  (16.7) | 57 (8.8) | 0 | 1  (0.7) | 1 (0.6) |
| **Number (%) of patients lost NEDA** | 79  (24.4) | 47  (15.0) | 5  (41.7) | 131 (20.2) | 2  (8.0) | 7  (5.1) | 9 (5.5) |

*Active MRI brain refers to the presence of a new and/or enlarging T2 inflammatory lesion and/or enhancing T1 inflammatory lesion on MRI brain. DMT = disease modifying therapy. NEDA = no evidence of disease activity. EDSS = expanded disability status scale.

**Supplementary Table 4. Reasons for patients not assessed for NEDA according to type of therapy.**

|  |  | **Glatiramer acetate**  **(N=342)** | **Dimethyl fumarate (N=335)** | **Fingolimod (N=12)** | **Natalizumab (N=26)** | **Ocrelizumab (N=138)** |
| --- | --- | --- | --- | --- | --- | --- |
|  |  | **non-HET**  **(689)** | | | **HET**  **(164)** | |
| **Not Assessed for NEDA** |  | 66 | 52 | 0 | 0 | 0 |
| **Reasons for stopping treatment early** | **Side effects** | 50 | 42 | 0 | 0 | 0 |
|  | **Moved trust and lost to follow up** | 12 | 8 | 0 | 0 | 0 |
|  | **Family planning** | 4 | 2 | 0 | 0 | 0 |

NEDA = No evidence of disease activity. Non-HET = non-high-efficacy therapy. HET = high-efficacy therapy.
